# Genomic engineering in *Rhizobium etli*: implementation and evaluation of systems based on dCas9

**DOI:** 10.3389/fmicb.2025.1604430

**Published:** 2025-06-24

**Authors:** Oussama Bellahsen, Rafael Díaz-Méndez, David Romero

**Affiliations:** Programa de Ingenería Genómica, Centro de Ciencias Genómicas, Universidad Nacional Autónoma de México, Cuernavaca, Mexico

**Keywords:** CRISPR-Cas9, CRISPRi, gene editing, gene repression, Rhizobiales

## Abstract

CRISPR-Cas9 is a powerful tool for gene editing and regulation, facilitating the analysis of gene function. In this study, we developed a robust CRISPR interference (CRISPRi) system to precisely modulate gene expression in the bacterium *Rhizobium etli*, the nitrogen-fixing symbiont of the common bean. The system is based on two compatible plasmids (pBBR1MCS2-dCas9 and pRhigRNA containing specific guide RNAs). Introduction of both plasmids in *R. etli* led to significant repression of four target genes [DsRedexpress, *recA*, *thiC* (on the *thiCOSGE* operon) and *rdsA*] depending on the guide RNA used. By employing different guide RNAs at various target sites, we obtained up to 90% gene repression. Importantly, neither significant secondary effects on growth nor toxicity were observed upon expression of dCas9, either alone or in co-expression with the guide RNAs. This system can be utilized for further investigations on the function of essential genes in *R. etli*, or it can be integrated with other gene expression elements or gene editing tools, such as base editors for advanced genome engineering in Rhizobiales.

## Introduction

Bacteria represent a fundamental part of life and its evolution. Understanding the functions encoded in their genomes involves a wealth of techniques and methods. One common approach is fine-tuning gene expression to understand its function, looking for specific phenotypes (Tong et al., 2023). Nowadays, remarkable advances in gene expression fine-tuning technologies enhance their usefulness for easier and more efficient regulation of the target gene, allowing a better understanding of the function of each target. These technologies can have several limitations, depending on the target gene, and/or the organisms to which it can be applied (Kent and Dixon, 2020). Clustered regularly interspaced short palindromic repeats interference (CRISPR-Cas) is one of the newest technologies in genome engineering. It was discovered in different bacteria and archaea as an adaptative immune system to prevent establishment of genomic invaders, such as phages and endogenous plasmids (Barrangou et al., 2022). The CRISPR-Cas system consists of a CRISPR array; a group of short repetitive sequences separating a large number of adapted short sequences called spacers, and a group of genes encoding the CRISPR accessory genes and the signature Cas protein (Wang and Doudna, 2023). As an immune system, the system employs mainly Cas1 and Cas2 to identify foreign DNA, searching for specific PAM sequences as a filter to select the target sequence to be acquired as a spacer, followed by insertion of the target sequence into the CRISPR array, which will afford protection against a specific invader (Varble and Marraffini, 2022). Once an invader enters the cell, the CRISPR array is transcribed and processed into a mature crRNA. Each crRNA binds to the Cas signature protein, forming a complex that recognizes the target through RNA–DNA interaction and cuts the invading DNA, leading to its degradation (Varble and Marraffini, 2022). With the discovery of different types of Cas systems, CRISPR-Cas9 (classified as type II), has become the system of choice for genome modification. This preference is due to the successful engineering of the system to a simpler version, based on the fusion of trans-RNA and crRNA into a single guide RNA, eliminating the need for multiple elements (Khan et al., 2022). Additionally, editing with CRISPR-Cas9 only requires the multidomain Cas9 protein, distinguishing it from other CRISPR-Cas types, in which operation of the system depend on several proteins (Khan et al., 2022). Each specific Cas9-sgRNA complex can detect an NGG PAM sequence and generate double-strand breaks (DSB) a few bases upstream of the PAM sequence (Khan et al., 2022). The subsequent repair of the DSB depends on the available DNA repair mechanisms, which can include non-homologous end joining (NHEJ) or homology-directed repair (HDR) systems (Verma and Greenberg, 2016; Sharda et al., 2020) The choice of repair mechanism determines the outcome of the DNA repair process, leading to an easy inactivation of the target gene.

Further modifications of the Cas9 system included the generation of a modified version of Cas9, known as dead Cas9 (dCas9), which harbors mutations in the two catalytic domains required for DSB generation. The system based on dCas9, referred to as CRISPRi, has become an efficient strategy for regulating gene expression (Khan et al., 2022). dCas9 retains the ability to scan DNA for a target and binds to it when the PAM sequence is detected, but it does not induce double-strand breaks (DSBs) in the DNA (Khan et al., 2022). Binding of dCas9 to sites encompassing the promoter or downstream elements lead to tight repression of the corresponding gene (Zhang and Voigt, 2018; Zhang et al., 2021) Additional modifications of the dCas9 system include fusion of dCas9 with a base editor, which can be successfully used to introduce specific point mutations (Zhang et al., 2020).

CRISPR-Cas9 has been highly successful for gene editing across numerous bacteria. Despite its success, toxicity and undesirable effects have been observed in several organisms for various reasons. However, these toxic effects were controlled or eliminated by achieving tight control of its expression through the use of regulated promoters or by incorporating additional elements to modulate the extent of expression of Cas9 (Soonsanga et al., 2020). Conversely, in other instances, conventional Cas9 systems for gene editing could not be adapted, but the cells could tolerate alternative versions of Cas9 such as Cas9 nickase or dCas9 (Xu et al., 2015; Luo et al., 2020).

*Rhizobium etli* is an alpha proteobacterium that is the nitrogen fixing symbiont of beans and is highly prevalent in Mexico (González et al., 2003). Given its significant agricultural importance to the region, extensive research has been conducted on the genetic basis of nodulation and nitrogen fixation, along with the fundamental characteristics of the species. *R. etli* has a complex genome, consisting of a main circular chromosome DNA and six large circular plasmids, one of which (p42e) is considered a secondary chromosome, due to its possession of two essential genes (Landeta et al., 2011). Although genetic manipulation techniques in *R. etli*, primarily based on conjugation, facilitate genome alterations such as plasmid addition or elimination, large deletions, and marker exchange, they have notable limitations. These include a low frequency of marker exchange, challenges for introducing specific single base changes, and difficulties in achieving controlled gene expression. These limitations are also shared by other Rhizobiales. Due to these limitations, implementation of a CRISPR-Cas systems would be very useful for genetic investigations not only in *R. etli* but for Rhizobiales in general. Previous work in our laboratory has succeeded in implementing a CRISPR-Cas9 system for Rhizobium that can achieve high efficiency knockouts of specific genes using NHEJ repair (Díaz-Méndez et al., 2025); other workers have implemented a CRISPR-mediated system for base editing in Rhizobium (Wang et al., 2021). In this work, we report the construction of a non-toxic dCas9 variant and its use in a CRISPRi system. Application of this system in four different positions within the *R. etli* genome (DsRed express, *recA*, *thiC* and *rdsA* genes), lead to marked reductions in gene expression levels (up to 90%); in most of the cases, the expected phenotype for each reduction was observed.

## Materials and methods

### Strains and media

Strains employed in this study are shown in Supplementary Table 1. *Escherichia coli* strains were cultured in Luria–Bertani (LB) medium (Sezonov et al., 2007) at 37°C. *R. etli* strains were grown at 30°C, either in Peptone Yeast Extract (PY) rich medium with the addition of calcium chloride (Noel et al., 1984) or in MMY minimal medium supplemented with biotin at 1 mg l^−1^ (Bravo and Mora, 1988). When needed, thiamin (2 μg ml^−1^) was also added to MMY medium. For antibiotic selection, rich media were supplemented with appropriate concentrations of antibiotics (in μg ml^−1^): fosfomycin, 75; gentamicin, 30; kanamycin, 30; nalidixic acid, 20; spectinomycin, 100 and tetracycline, 10.

### Genetic procedures

For introduction of plasmids into *E. coli*, cells competent for transformation were prepared with a RbCl_2_-CaCl_2_ procedure, described previously (Green and Rogers, 2013). Transformed cells were plated on LB agar containing the appropriate selection antibiotic and incubated for 24 h at 37°C. The resulting colonies were selected and screened for the presence of the desired construct.

Introduction of plasmids into *R. etli* (either pBBR1MCS-2 dCas9 or pRhigRNA containing appropriate guides, see below) was done by biparental plate matings. For that, plasmids were transformed into *E. coli* strain S17-1 (Simon et al., 1983). Transformed *E. coli* cells were then used as donors for conjugation. For each conjugation, *E. coli* S17-1 was grown overnight in LB medium containing the appropriate antibiotic, while *R. etli* was grown in PY medium supplemented with the corresponding antibiotic. After 24 h of growth, both cultures were centrifuged, washed with sterile water and resuspended in 1 mL of antibiotic-free PY medium. Mixtures composed of 700 μL of *R. etli* (OD = 1.1) suspension (recipient) and 350 μL of S17-1 (OD = 1.5) suspension (donor) were vortexed, spun down, and the resulting pellet was resuspended in 150 μL of PY medium. This suspension was deposited onto a plate containing solid PY medium without antibiotics and incubated at 30°C for 24 h. Following incubation, the bacterial growth was scraped off the plate and resuspended in liquid PY medium. Serial dilutions were plated on PY medium containing the appropriate antibiotic to select for introduction of the desired plasmid. Selected colonies were verified for the presence of the desired plasmid using Eckhardt gel electrophoresis (Eckhardt, 1978) as modified by Hynes and McGregor (1990). This technique is based on an in gel lysis of the cells followed by separation in a conventional agarose gel, allowing separation of circular molecules ranging in size from 100 to 2,500 kb. When appropriate PCR amplification with specific primers was also carried out.

### Construction of a plasmid with dCas9

For construction of a catalytically inactive variant of Cas9 (dCas9) specific point mutations were introduced into both catalytic domains (RuvC and HNH), using a two-step PCR procedure. To achieve this, a 97 bp megaprimer containing the D10A mutation was amplified using primers M1 and M2 and plasmid pRhiCas9 (a pBBR1MCS-5 derivative harboring Cas9 under the control of a *lacZ* promoter, Díaz-Méndez et al., 2025) as a template (Supplementary Table 2). The resulting PCR product harboring the D10A mutation was then used as a megaprimer along with a third primer (M3) and pRhiCas9 as a template to amplify a larger fragment (2.6 Kb) carrying the RuvC mutation. The resulting PCR product (RuvC*) and plasmid (pRhiCas9) were digested with KpnI and AatII, gel purified and ligated, thus creating a plasmid (pRhi-nCas9) containing a nCas9 (RuvC*) variant. Next, the second mutation (inactivating the HNH catalytic domain, H840A) was generated by PCR amplification using pRhi-nCas9 as a template and two different primers (M4 and M5), one of which harbors the H840A mutation (Supplementary Table 2). The resulting 1.68 Kb PCR product and plasmid pRhi-nCas9 were digested with XhoI and AatII, gel-purified and ligated, facilitating the replacement of the original fragment with the mutated HNH* fragment. This process generated a plasmid pRhi-dCas9 (pBBR1MCS-5) carrying the dCas9 sequence with mutations in both catalytic domains, RuvC* and HNH*. The resulting dCas9 sequence, along with the original full promoter cassette, was transferred into a similar plasmid (pBBR1MCS-2) carrying Km resistance. To achieve this, both pRhi-dCas9 (pBBR1MCS-5) and the new vector (pBBR1MCS-2) were digested with AgeI and XhoI, and gel-purified. The purified dCas9 sequence along with the full promoter cassette was then ligated into the open pBBR1MCS-2 vector, generating a new dCas9 construct (pBBR1MCS-2 dCas9). To reduce the incidence of unwanted mutations, all PCR reactions were performed using a Phusion High-Fidelity DNA Polymerase Master Mix (Thermo Scientific), following the instructions of the manufacturer. The full gene for dCas9 was sequenced.

### Guide RNA design

The guide RNAs (Supplementary Table 3) were designed using Protospacer Workbench (MacPherson and Scherf, 2015) and the genomic sequence for *R. etli* CFN42 (GCF_000092045.1) from NCBI, or a custom database for *R. etli* CFN42 containing additional genetic elements, such as the insertion of Red fluorescent protein gene (DsRedexpress). Protospacer Workbench was used in the default mode, looking for guide RNAs predicted to lack off-target effects. DNA fragments encoding the guide RNAs for specific regions were synthesized as pairs of complementary primers, with four extra-base pairs matching the protruding ends for BbsI, annealed and cloned into BbsI-restricted pRhigRNA plasmid. To confirm successful cloning, both PCR amplification with specific primers and sequencing were performed for all cloned constructs. Relevant sequencing primers are listed in Supplementary Table 2.

### Phenotypic screenings

To evaluate levels of fluorescence, cells of *R. etli* with the DsRedexpress gene and their derivatives were grown overnight in a liquid PY medium containing the appropriate antibiotic at 30°C. When needed, IPTG (Isopropyl ß-D-1-thiogalactopyranoside) was used at 0.8 mM. Overnight cultures were washed with sterile water and used to inoculate 96-well plates containing PY medium, at an initial A_600_ of 0.05. The 96-well plates were incubated and registered in a Synergy HT reader, configured to capture cell density (A_600_) and dsRed express cell fluorescence (excited at 558 nanometers, with an emission maximum at 583 nanometers), with hourly measurements over a 42-h period. Fluorescence values were normalized according to the A_600_ values, and the set of values for each condition was graphed for determination of area under the curve (AUC). Statistical analyses were performed with the AUC values as described below. Three biological replicates, each with three technical replicates, were done for every condition and strain.

For experiments aimed to achieve repression of *recA*, sensitivity of the strains to UV light was also evaluated. All experiments were started from *R. etli* cells from frozen glycerol stocks, which were cultured for 48 h in liquid PY-medium. Cells were then used to start cultures in fresh PY medium supplemented with the necessary antibiotics for an additional 24 h. Following growth, each culture was diluted and divided into three fractions. Two fractions were exposed to different UV fluences (5 and 10 J/m^2^) using a Hoefer UVC 500 Ultraviolet Crosslinker, while the third fraction was left unexposed. After that, each fraction was plated onto solid media containing the appropriate antibiotics and incubated in darkness at 30°C for 4 days. After incubation, the resulting colonies were analyzed and counted to determine the percentage of surviving cells under each condition.

In experiments involving inhibition of *thiC* expression, thiamin auxotrophy was evaluated following a protocol designed to minimize thiamin carryover from cells grown in rich medium. For that, *R. etli* cells were cultured overnight in PY medium supplemented with the appropriate antibiotics. Subsequently, they were washed and grown in MMY medium for 24 h, with the necessary antibiotics. Resulting cells were washed with MMY, diluted, and plated onto plates solidified with agarose containing two different media without antibiotics: one with standard MMY and the other with MMY supplemented with thiamin. Plates were incubated at 30°C for 4 days, recording colony size and number.

### Microscopy

For microscopical analyses, *R. etli* cells were grown overnight in liquid PY medium with the appropriate antibiotics. Cells were used to inoculate fresh liquid cultures in MMY medium without antibiotics for 12 h. Slides using 1% agar and MMY medium were prepared, and 40 μL of each culture were deposited; slides were left drying until the bacteria were fixed. To assess cell morphology, we employed a Nikon Ti-E inverted microscope equipped with Nikon’s perfect Focus system and a motorized stage. The experiment was performed using NIS-elements 4.20 AR software to examine all slides using a 100x Plan APO objective to ensure the best field for clear imaging. Bright-field images were captured using a camera connected to the microscope, with pictures taken from various regions of the slides.

### Quantitative RT-qPCR analysis

To quantify gene expression levels for all dCas9 targets, nucleic acids were extracted using a standardized TriZol protocol. Samples were treated with DNAse and checked via PCR to ensure the RNA’s purity (DNA-free stocks). Subsequently, the RNAs were reverse transcribed into cDNA using cDNA Synthesis Kit (Thermo Scientific) for utilization in real-time PCR quantification. Real-time PCR runs employed specific primers that were designed to amplify 200 base pairs of each target (Supplementary Table 2). Each reaction was executed in a 15 μL volume: 7.5 μL of SYBR Green master mix, 0.75 μL of each primer adjusted to 10 pmol/μl (listed in Supplementary Table 2), 4 μL of H_2_O, and 2 μL of template DNA (1,000 ng/μl). The runs were performed using a StepOne Real-Time PCR instrument, with a specific software version (v2.2.2), with an initial denaturation step at 95°C for 10 min, followed by a thermal cycling regime composed of 40 cycles of denaturation at 95°C for 15 s and annealing/extension at 60°C for 1 min. Following the 40 cycles, a melting peak analysis was performed from 60°C to 95°C with increments of 0.1°C, concluding the run with a cooling step to 4°C. Subsequently, the results were collected, and quantification ratios were calculated based on the cycle threshold (CT) values. Three biological replicates, each with three technical replicates were conducted for every condition and strain.

### Statistical analysis

In this study, we statistically analyzed qRT-PCR data to assess the significance of differences in gene expression levels between the tested and control samples, using GraphPad Prism software. First, we verified that the data met all the necessary requirements for the proper analysis. Thus, we applied a one-way ANOVA followed by Tukey’s multiple comparisons test. This approach allowed us to determine whether significant differences existed between the mean expression levels of the three biological replicates of the control and those of the other tested samples.

## Results

### Construction of a dCas9 derivative useful for CRISPRi in *Rhizobium etli*

**Figure 1:**
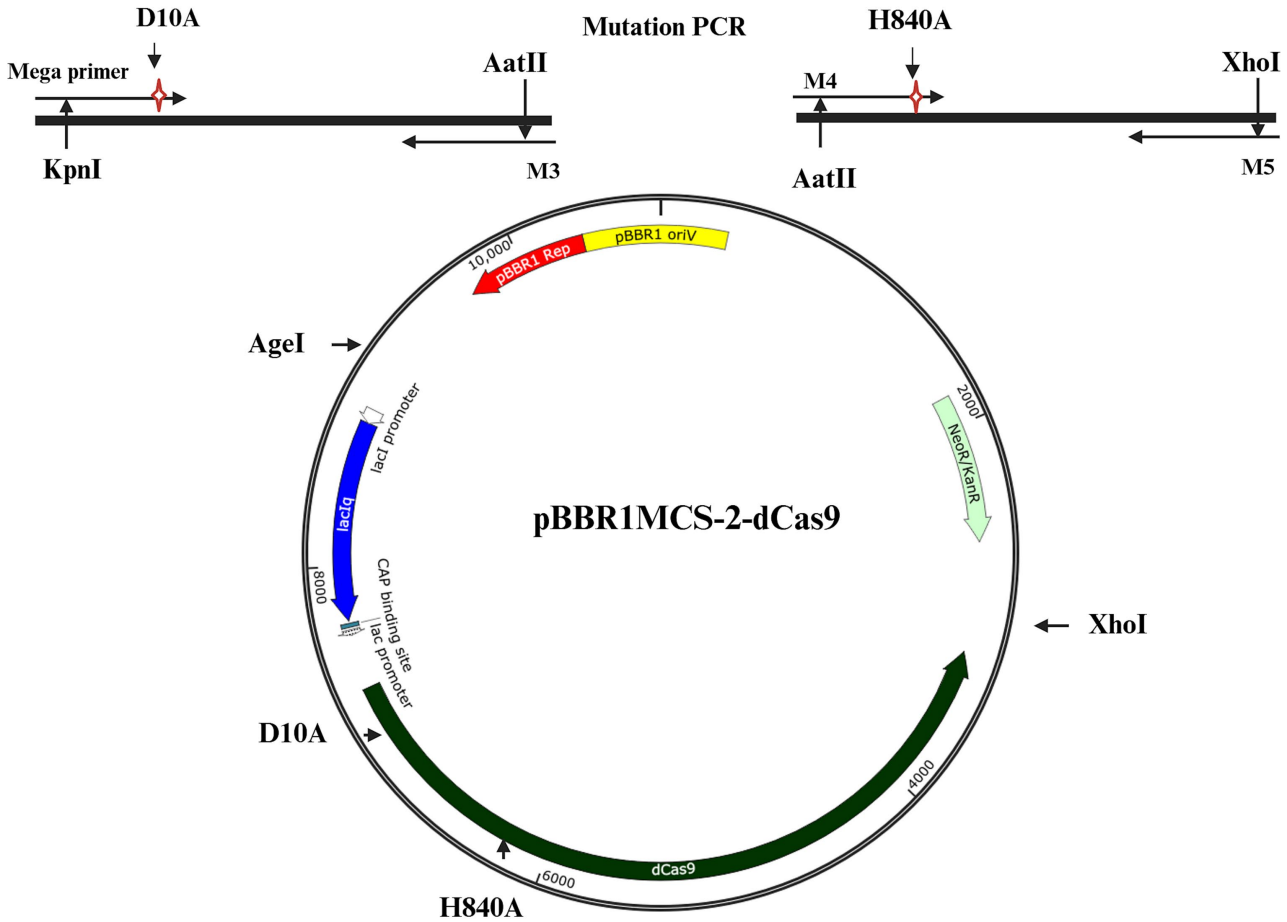
Schematic representation of the plasmid harboring dCas9. The required mutations to generate dCas9 were introduced by PCR (see Materials and Methods) in two separate fragments (top part) which were combined to generate dCas9. The whole construct was integrated on a pBBR1MCS-2 backbone, to generate pBBR1MCS-2-dCas9.

To finely regulate gene expression in *R. etli*, we devised a system, based on the use of two compatible plasmids of medium (5 to 8) copy number. The first plasmid, called pRhigRNA, is a pRK415 derivative (Keen et al., 1988) containing a region transcribed from a *lacZ* promoter, useful to generate appropriate guide RNAs directed at specific genomic targets (Díaz-Méndez et al., 2025). The second plasmid was constructed in this work and is a pBBR1MCS-2 (Kovach et al., 1995) derivative containing a gene for *Streptococcus pyogenes* dCas9 (Spy dCas9). This dCas9 was generated by introducing two mutations, D10A and H840A, effectively disabling both catalytic domains (Materials and Methods). Subsequently, the modified dCas9 was integrated into the pBBR1MCS-2 vector, under the control of a *lac* promoter, giving rise to pBBR1MCS-2 dCas9 (Figure 1). Introduction of the desired mutations was confirmed by sequencing. Introduction of both plasmids into *R. etli* should be enough to achieve gene repression of specific genomic targets through CRISPRi. Since expression of both dCas9 and the guide RNA originate from lac promoters, the degree of inactivation might be dependent on the control of this promoter.

### DsRedexpress gene repression: evaluation of an effective CRISPRi system

Initial tests for operation of CRISPRi employed a *R. etli* strain in which the Red fluorescent protein gene (DsRed-express) was integrated into the chromosome. Integration was done using a Mini-Tn7 [mini Tn7 (Gm) P_A1/04/03_ DsRedExpress-a, (Lambertsen et al., 2004)] ensuring no interference with the native gene expression (Torres Tejerizo et al., 2015). It was reported that expression of DsRedExpress is dependent on a modified *lacZ* promoter [P_A1/04/03_ promoter, (Lambertsen et al., 2004)], so a guide RNA was designed matching the sequence of this promoter and cloned into pRhigRNA. Unexpectedly, upon introduction of both pRhigRNA and pBBR1MCS-2 dCas9 into *R. etli* DsRedExpress, no reduction in red fluorescence was detected (data not shown). To verify that the sequence of the designed guide RNA matched with the promoter controlling DsRedExpress, the corresponding sequence in *R. etli* DsRedExpress was amplified and sequenced (see Materials and Methods). Sequence analysis (Supplementary Figure 1) revealed that, contrary to reported data (Lambertsen et al., 2004), the DsRedExpress gene is not controlled by a P_A1/04/03_ promoter, but with the *E. coli* growth-regulated promoter *rrnB P1*.

**Figure 2:**
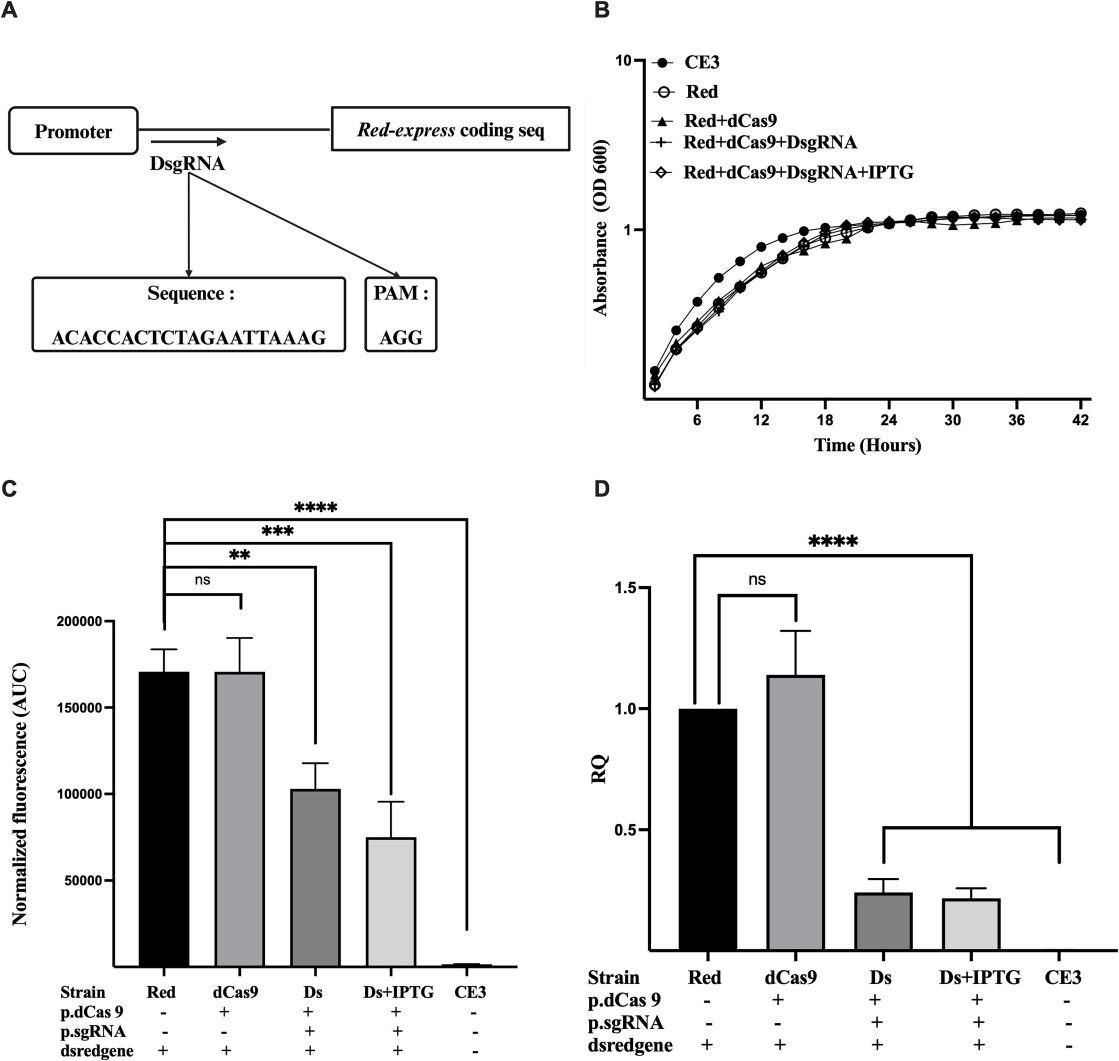
Downregulation of red protein gene (DsRedexpress) expression by CRISPRi. **(A)** Schematic overview of the guide RNA (DsgRNA) positioned downstream of the Dsred-expression promoter, the precise sequence, and the associated PAM sequence. **(B)** Growth kinetics of *R. etli* strains CE3 (wildtype), Red (CE3 strain labeled with Tn7 Dsred-express), Red+dCas9 and Red+dCas9 + DsgRNA. Addition of the inducer (IPTG) is indicated. **(C)** Fluorescence levels for each strain, expressed as area under the curve (AUC). For AUC calculation, fluorescence levels were recorded at different times of the growth cycle (42 h), normalized according to growth absorbance (Supplementary Figure 2). **(D)** Relative quantification of Dsred-express gene expression in the different strains was measured after 24 h of growth in PY rich medium by qRT-PCR. Data in panels B to D are means (+/− SD) of at least three independent determinations for each strain. Results were analyzed statistically by one-way ANOVA followed by Tukey’s multiple comparisons test. NS, (not significant, *p* > 0.05); ** (*p* < 0.002); *** (*p* < 0.0002); **** (*p* < 0.0001). Location of the region targeted by the guide RNA with respect to the promoter and the coding sequence is shown in Supplementary Figure 3.

Having identified the correct promoter sequence controlling *DsRedExpress*, we designed a new DsgRNA, matching the region between the −10 and the start codon of the Red fluorescent protein (Figure 2A; Supplementary Figure 3), to be used with dCas9. These positions were chosen as the most likely to lead to strong repression, based on the experience in other systems (Bikard et al., 2013; Larson et al., 2013) There were no notable effects observed on cell growth across all conditions, whether dCas9 was expressed alone or in conjunction with its respective guide RNA (Figure 2B). The results showed that a strain co-expressing dCas9 and DsgRNA (strain Ds) exhibited a significant reduction in fluorescence intensity compared to the *R. etli* DsRedexpress strain (lacking any CRISPR construct), irrespective of IPTG presence. This stands in contrast to a strain expressing only dCas9, which exhibited a fluorescence level indistinguishable from the one seen in the *R. etli* Red strain (Figure 2C). Following these observations, an analysis of relative quantification of transcripts (RQ), done by qRT-PCR (Figure 2D), demonstrated a significant decrease in gene expression (up to 80%), upon co-expression of dCas9 and DsgRNA (strain Ds), independent of IPTG induction, compared to the Red strain. In the Red+dCas9 strain, where no guide RNA was expressed, *DsRed-express* gene expression was similar to that of the Red strain, showing no significant differences (Figure 2D).

Both qRT-PCR and phenotypic analyses corroborated that dCas9 effectively suppressed *DsRed-express* expression only in the presence of the appropriate guide RNA (DsgRNA). Maximal repression levels were achieved even without IPTG induction, suggesting that transcriptional escape from the lac promoter is enough to provide adequate levels of dCas9 and the guide RNA to instrument CRISPRi. Moreover, no significant effects on growth were observed upon expression of both dCas9 and DsgRNA, suggesting the absence of toxic effects for CRISPRi in *R. etli*. To evaluate if unwanted effects on cell morphology occur in the presence of CRISPRi, strains were analyzed by light microscopy. Cells from the parental Red strain displayed a normal rod shape without significant levels of filamentous or spherical cells (Supplementary Figure 4). Normal morphology was also seen when cells expressed dCas9 or both dCas9 and DsgRNA, even in the presence of IPTG (Supplementary Figure 4). These results rule out unwanted effects on cell growth upon CRISPRi in *R. etli*.

To further ascertain that the observed reductions in red fluorescence were due to CRISPRi, both pBBR1MCS-2 dCas9 and pRhigRNA/ DsgRNA were readily eliminated from the Red strain by five cycles of subculturing in the absence of antibiotics. As expected, the strain lacking these plasmids recover normal fluorescence levels (Supplementary Figure 5). These results confirm the successful implementation of a CRISPRi system for *R. etli*.

### CRISPRi of the *recA* gene phenocopies the effects of a *recA* mutation

After demonstrating that CRISPRi can suppress the expression of an exogenous gene added to the chromosome of *R. etli*, we decided to explore if it works on a naturally accrued chromosomal gene. For that, the *recA* gene was chosen as a target. Besides its effects on DNA recombination and repair, the RecA protein plays a crucial role in regulating the SOS response. Upon encountering DNA damage, the apoprotease activity of RecA is activated, catalyzing its own cleavage and the cleavage of LexA, the repressor of the SOS system. Lack of *recA* expression provokes a marked sensitivity to DNA-damaging agents (Martinez-Salazar et al., 1991; Martínez-Salazar et al., 2009).

**Figure 3:**
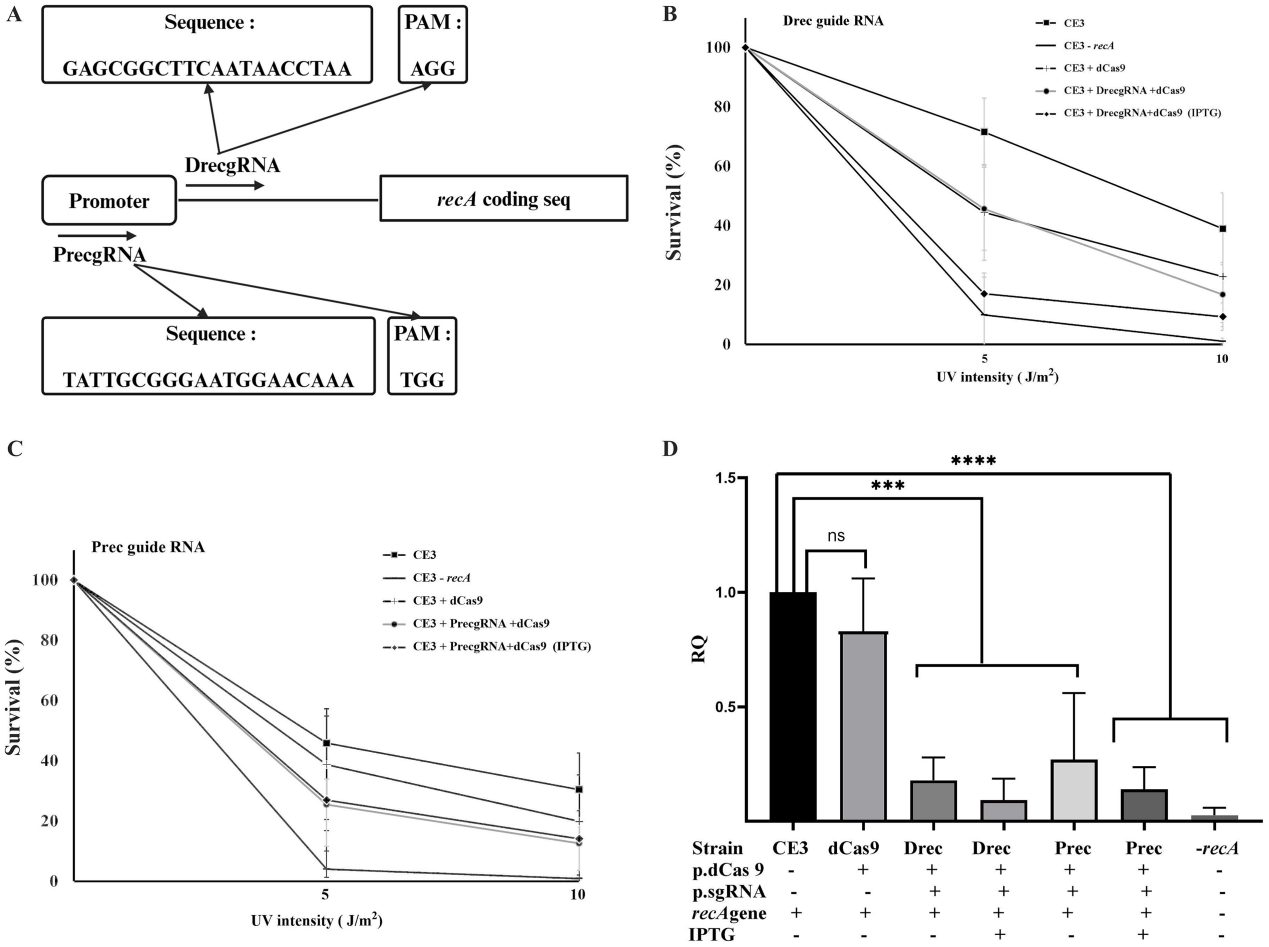
CRISPRi of *recA* gene expression. **(A)** schematic representation of the location of the two guide RNAs used (DrecgRNA and PrecgRNA) relative to the promoter of the *recA* gene. **(B)** and **(C)** effect of the expression of dCas9, either in the presence or in the absence of the indicated gRNAs, on the sensitivity of *R. etli* strains to UV irradiation. For the two exposition levels, data are expressed as the surviving fraction relative to the unexposed condition. **(D)** Relative quantification of *recA* gene expression levels was done after 24 h of growth in PY rich medium by qRT-PCR. Data in panels B to D are means (+/− SD) of at least three independent determinations for each strain. A *R. etli recA* mutant strain is included as a control. Results were analyzed statistically by one-way ANOVA followed by Tukey’s multiple comparisons test. NS, (not significant, *p* > 0.05); *** (*p* < 0.0002); **** (*p* < 0.0001). Location of the regions targeted by the guide RNAs with respect to the promoter and the coding sequence is shown in Supplementary Figure 3.

To evaluate the impact of CRISPRi on the *R. etli recA* gene, two different guides were designed (Figure 3A; Supplementary Figure 3): one (PrecgRNA) complementary to the −35 region of the promoter and the SOS box and another (DrecgRNA) matching a sector between the transcriptional start site and the start codon of *recA* (Tapias et al., 1997; Tapias and Barbé, 1998) Upon joint expression of dCas9 and suitable guide RNAs, a marked sensitivity to UV irradiation was observed (Figures 3B,C). The results indicate that cells co-expressing dCas9 along with DrecgRNA (Figure 3B) or PrecgRNA (Figure 3C) guide RNAs displayed higher sensitivity to UV radiation, resembling the one seen in a *recA* mutant (CE3:*recA*). However, the same strain expressing only dCas9 without any of the guide RNAs displayed UV sensitivity less significant and closely similar to the wild-type strain (Figures 3B,C). These phenotypes suggest that the downregulation of *recA* expression renders cells more susceptible to UV damage. To evaluate the impact of CRISPRi on *recA* expression quantification of transcripts was done by qRT-PCR (Figure 3D). Data show that the strain containing only dCas9 exhibited *recA* expression levels like the wild type. In contrast, strains coexpressing dCas9 with either DrecgRNA or PrecgRNA reveal significant reductions in *recA* gene expression (ca. 90%), even in the absence of IPTG induction. The observed downregulation of *recA* expression upon CRISPRi is consistent with the enhanced sensitivity of these strains to DNA damage. These results validate the functionality of CRISPRi to reduce the expression of a chromosomal gene in *R. etli*.

### Inhibition of a plasmid-located thiamin biosynthetic operon

**Figure 4:**
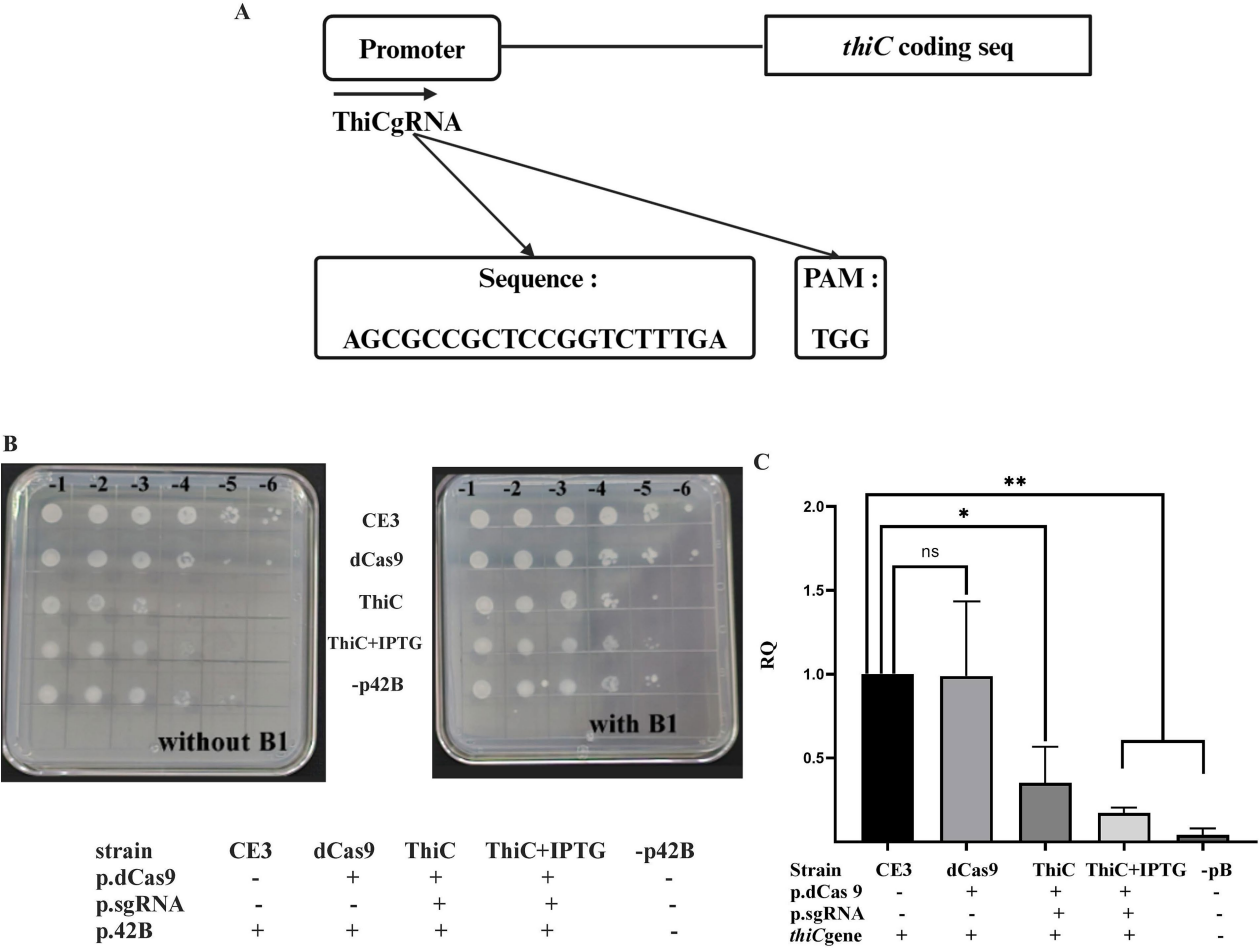
*thiC* gene repression: **(A)** Schematic representation of ThiCgRNA designed on top of the promoter of the *thiC* gene. **(B)** effect of the expression of dCas9, either in the presence or in the absence of the specific gRNAs, on growth. All strains were initially grown in liquid MMY minimal medium to saturation (ca. 10^8^ cells per ml) and appropriate dilutions were spotted onto solid MMY in the absence or in the presence of vitamin B1, as indicated. Two biological repetitions were done. **(C)** Relative quantification of *thiC* gene expression levels across the considered strains was done as described in Figure 2. Data in panel C are means (+/− SD) of at least three independent determinations for each strain. A *R. etli* strain lacking plasmid p42b is included as a control. Results were analyzed statistically by one-way ANOVA followed by Tukey’s multiple comparisons test. NS (not significant, *p* > 0.05); * (*p* < 0.03); ** (*p* < 0.002). Location of the region targeted by the guide RNA with respect to the promoter and the coding sequence is shown in Supplementary Figure 3.

To determine if the CRISPRi system should be useful to reduce expression of genes located in the endogenous plasmids of *R. etli*, we decided to target the *thiCOSGE* operon located on plasmid p42b (184 kb). The *thiCOSGE* operon is required for *de novo* thiamin biosynthesis in *R. etli* (Miranda-Ríos et al., 1997, 2001) loss of expression of this operon do not lead to a complete auxotrophy due to the presence of a thiamin salvage pathway (*thiMED*) located on a separate plasmid, p42e (Karunakaran et al., 2006) To inhibit the expression of the *thiCOSGE* operon a guide RNA was designed (ThiCgRNA, Figure 4A; Supplementary Figure 3), matching the −35 region of the promoter of the operon and the intervening region toward the −10 (Ramírez-Romero et al., 2006); the guide RNA was introduced into *R. etli* in conjunction with dCas9. As shown in (Figure 4B), joint expression of ThiCgRNA and dCas9 instigate reduced cell growth in the absence of vitamin B1, at levels similar to those observed in a strain lacking the *thiCOSGE* operon (strain lacking p42b). The effect on growth in a strain expressing dCas9 and ThiCgRNA was not augmented upon inducing the system (i. e. upon IPTG addition). Normal growth was recovered in all cases upon vitamin B1 supplementation. No effects on cell growth were seen when only dCas9 was introduced in cells.

To quantify the magnitude of CRISPRi on the expression of the operon, qRT-PCR analyses were conducted, evaluating the expression of *thiC*, the first gene of the operon. Data show that expression of dCas9 and ThiCgRNA led to a reduction in *thiC* gene expression by approximately 60% in the absence of IPTG induction, which increased to around 80% upon induction with IPTG. No significant effects on *thiC* gene expression were observed when only dCas9 was expressed within the cell (Figure 4C). These results demonstrate that CRISPRi can be used successfully with genes located on endogenous plasmids.

### CRISPRi of the *rdsA* essential gene

**Figure 5:**
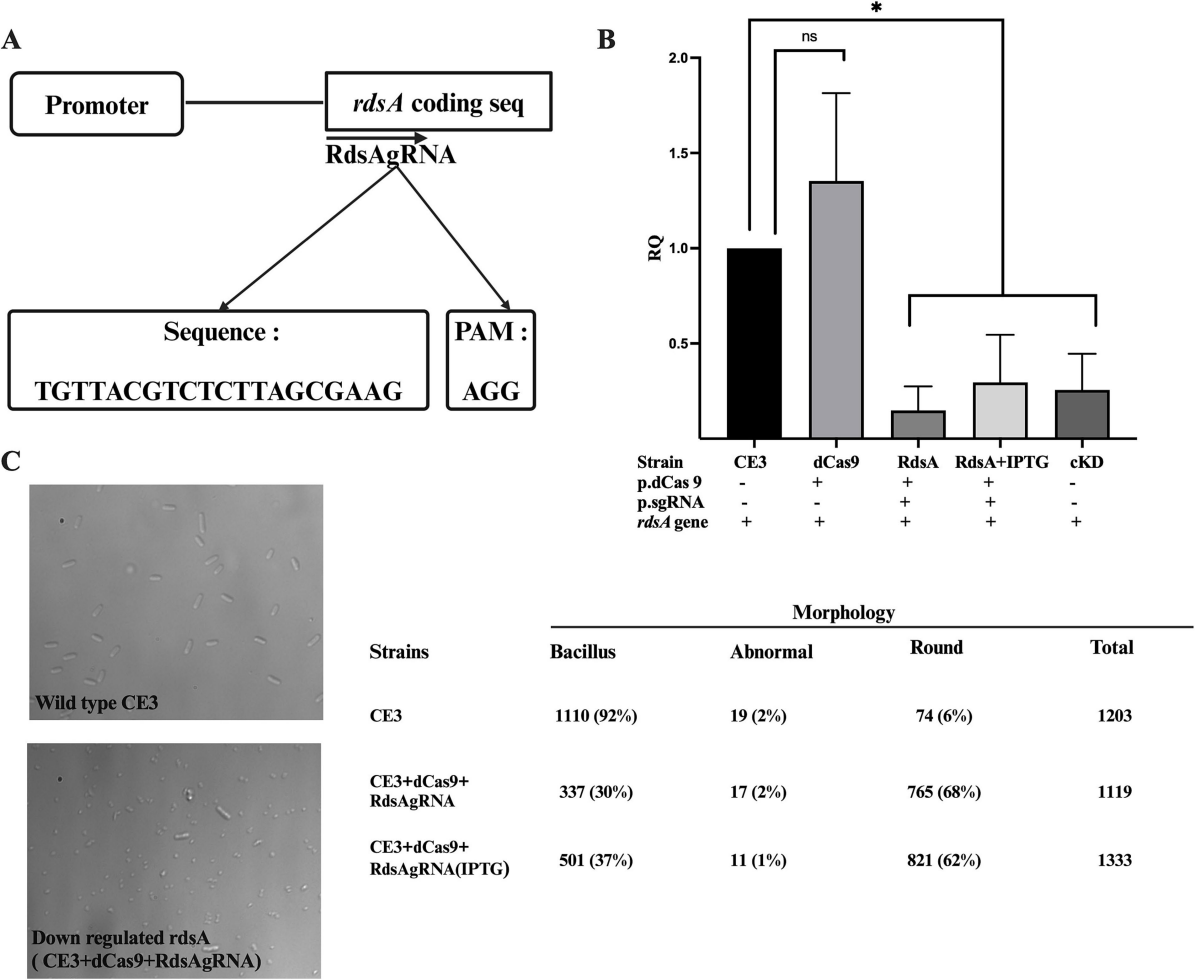
CRISPRi of the *rdsA* gene affects cell morphology. **(A)** Schematic representation of RdsAgRNA designed at the beginning of the coding sequence of *rdsA*, with the appropriate complementary sequence and its PAM. **(B)** Relative quantification of *rdsA* gene expression levels across the indicated strains was done as described in Figure 2. Data are means (+/− SD) of at least three independent determinations for each strain. A *R. etli* strain having a conditional knockdown in *rdsA* gene expression (cKD24; Martínez-Absalón et al., 2022) was included as a control. Statistical analyses were carried out to assess the significance of differences among all relative quantification (RQ) values. Results were analyzed statistically by one-way ANOVA followed by Tukey’s multiple comparisons test. NS, (not significant, *p* > 0.05); * (*p* < 0.03). **(C)** Microscopical analysis of the wild-type strain (CE3) and the CRISPRi *rdsA* strain (CE3 + dCas9 + RdsAgRNA in the presence of IPTG). Counting of the different cell morphologies for each strain (*n* > 1,000 cells) was done. *Bacillus*, normal rod-shaped cells; abnormal, Branched cells or cells with more than one growth foci; Round, spherical cells. Location of the region targeted by the guide RNA with respect to the coding sequence is shown in Supplementary Figure 3.

To demonstrate the application of the CRISPRi with an essential gene, we decided to target gene *rdsA* (RHE_PE00024), a gene located on the plasmid p42e of *R. etli*. The *rdsA* gene plays a critical role in determining *R. etli* cell division and shape (Martínez-Absalón et al., 2022). It acts as a key transcriptional regulator, influencing multiple biological processes such as cell division, shape, and peptidoglycan biosynthesis, either directly or indirectly (Martínez-Absalón et al., 2022). Being an essential gene, knockout mutants cannot be produced, but knockdown mutants, where *rdsA* was put under the control of a cumate-inducible promoter, displayed a high number of round cells, instead of the normal rod shape (Martínez-Absalón et al., 2022). A specific guide RNA was designed (RdsAgRNA) matching a sector encompassing the beginning of the *rdsA* coding sequence (Figure 5A; Supplementary Figure 3). Quantification of *rdsA* transcript levels by qRT-PCR demonstrated a 80% reduction in *rdsA* gene expression when both dCas9 and RdsAgRNA were co-expressed (Figure 5B). This reduction in expression was similar to the levels seen in a knockdown mutant in *rdsA* (Martínez-Absalón et al., 2022), grown in the absence of cumate. As expected, no significant reduction in *rdsA* transcript levels was observed in a strain expressing only dCas9 (Figure 5B).

Observation of these strains by light microscopy revealed a high percentage of round-shaped cells (up to 68%) in the strain expressing jointly dCas9 and RdsAgRNA, regardless of IPTG complementation (Figure 5C). These results closely match the ones seen for a *rdsA* knockdown mutant (Martínez-Absalón et al., 2022). Thus, the CRISPRi system can also be applied to identify and study essential genes.

## Discussion

In this work, we report the successful implementation of a CRISPRi system for use in Rhizobiales. This system allows for easier and more efficient study of unknown genes, targeting both essential and non-essential genes without introducing any permanent mutations. Guide RNAs and dCas9 are provided in separate, compatible plasmids, wherein expression of the elements of the system are controlled through a *lacZ* promoter. Toxicity of the components of CRISPRi in some organisms (Vento et al., 2019) has limited the application of this approach. For instance, in *E. coli* high levels of dCas9 alone lead to drastic filamentation (Cho et al., 2018). In other bacteria, Cas9 expression was lethal to the cell (Vento et al., 2019). However, separate introduction of these elements in *R. etli* do not provoke any detectable toxicity on the cells, neither in survival nor in growth rate. We show that CRISPRi provoke specific reductions in expression in four target genes, two located on the chromosome (DsRedExpress-a and *recA*) and two located on large plasmids (*thiC* in p42b and *rdsA* on p42e). Consistent with the observed reductions in gene expression, CRISPRi strains display the expected phenotypes for knockout or knockdown mutations in the corresponding genes. Thus, CRISPRi can be applied for any of the replicons in the rhizobial genomes. Moreover, as demonstrated in the case of *rdsA*, CRISPRi can be successfully applied for essential genes. In those bacteria in which CRISPRi-dCas9 has been used extensively in genome-wide screens for essential genes, such as *E. coli* (Rousset et al., 2018), *Bacillus subtilis* (Peters et al., 2016) and *Vibrio cholerae* (Caro et al., 2019), repression levels analogous the ones reported here were adequate to validate gene essentiality.

Regarding similar systems in other soil bacteria, there is a CRISPRi system in *Sinorhizobium meliloti*, based on a different Cas, Cas12k (Cui et al., 2022). The authors only check the functionality of the system using GFP, achieving inhibition at levels like the ones described here, but they did not check the effect of the system on native chromosomal or plasmid genes. Working with *Pseudomonas putida* (Batianis et al., 2020) reported a CRISPRi-dCas9 system, with gene repression efficiencies comparable to the ones seen by us. Weaker repression efficiencies have been reported for *Azotobacter vinelandii* (Russell et al., 2024).

Although expression of both guide RNA and dCas9 is controlled with *lac* promoters, marked reductions in gene expression (60–90%) were achieved even in the absence of IPTG induction. These data suggest that transcriptional escape from *lac* promoters is enough to instrument repression. Leakiness in expression of either Cas9 or dCas9 is a problem that appear almost in every system tested. It is present even in CRISPRi systems for *B. subtilis* (Peters et al., 2016). It has been reported that tight control of the *lacZ* promoter in Rhizobiales is difficult to achieve, especially in medium-copy number plasmids (Khan et al., 2008). Transcriptional leakage has been observed for other inducible promoters (*Para*, *PrhaA*, *PmelA*, and *Ptau*) in *Sinorhizobium meliloti* (Mostafavi et al., 2014). Similarly, basal expression was reported for the *pvirB* promoter in *A. tumefaciens*, used in a CRISPR-based genome editing system (Rodrigues et al., 2021). Thus, to improve the control of the CRISPRi system described here, we are considering different alternatives, including chromosomal integration of dCas9 (using a miniTn7 derivative), incorporate additional regulatory elements, and/or switch to a different promoter.

A promising approach would entail the addition of a riboswitch to our dCas9 construct. Riboswitches are natural RNA aptamers that can form secondary structures, preventing translation; loss of this secondary structure can be achieved in the presence of a specific inducer, allowing translation of the transcript (Topp et al., 2010; Hallberg et al., 2017). By using a riboswitch, we can achieve tight control over dCas9 expression, ensuring that escape transcripts from the lac promoter remains untranslated due to the riboswitch’s secondary structure. This approach has demonstrated its utility for work with essential genes in *A. tumefaciens* (Grangeon et al., 2017). Another alternative is to insert a self-splicing group I intron at the beginning of a transcript, where splicing is regulated by the addition of an aptamer. This alternative, implemented in the SIBR-Cas system (Patinios et al., 2021), has proven useful to control basal expression of Cas12a.

It is conceivable that the fine-tuning of gene expression with CRISPRi in the free-living phase of *R. etli* can be extended to control gene expression when *R. etli* is in symbiosis. To achieve this, stability of the elements of the CRISPRi system during the symbiotic phase must be ensured, either through chromosomal integration or by adding elements that confer stability to the plasmids such as *parAB* sequences (Weinstein et al., 1992). It has been shown that the *lac* promoter and the repressor gene *lacI* can control rhizobial genes even during nodule development (Box and Dale Noel, 2011). An optimized concentration of IPTG was sufficient to activate gene expression during symbiosis, and with a rinsing protocol, it was possible to repress its expression (Box and Dale Noel, 2011). Thus, we can use the existing dCas9 construct to control gene expression of *R. etli* during symbiosis. Alternatively, different promoters that have proven effective for gene expression mainly during symbiosis can be employed, such as the *nodA* (Collavino et al., 2005) and the *nifH* (Bañuelos-Vazquez et al., 2019) promoters. Current work is devoted toward this objective.

## Data Availability

The raw data supporting the conclusions of this article will be made available by the authors, without undue reservation.
