# Supplementary material for: Genomic engineering in Rhizobium etli: implementation and evaluation of systems based on dCas9
Source: Front Microbiol. 2025 Jun 24;16:1604430. doi: 10.3389/fmicb.2025.1604430 (PMC12236228; doi:10.3389/fmicb.2025.1604430)
Supplement: Supplementary file 1 [file Data_Sheet_1.pdf]

Supplementary Material

1 Supplementary Figures and Tables

CAATTCGTTCAAGCCGAGATCGGCTTCCCGGCCGCGGAGTTGT

TCGGTAAATTGTCACAACGCCGCGGCCAATTCTAATTGGGGAC

CCTAGAGGTCCCCTTTTTTATTTTAAAAATTTTTTCACAAAAC

GGTTTACAAGCATAAAGCTTGCTCAATCAATCACCGGATCCCG

ACGGGCCCGGTACCGCGGCCGCGCGAATTCGAGCTCGTTGCGC

GGTCAGAAAATTATTTTAAATTTCCTCTTGTCAGGCCGGAATA

-35

ACTCCCTATAATGCGACACCACTCTAGAATTAAAGAGGAGAAA

-10

TTAAGCATGCGGTCTTCCAAGAATGTTATCAAGGAGTTCATGA

Coding sequence (Dsred-express)

Supplementary Figure 1. Sequencing of the promoter region of mini Tn7 (Gm) $P_{A1/04/03}$ DsRedExpress-a. Relevant region was amplified by PCR from *Rhizobium etli* Red strain using primers Fw-red and Rev-Gm and sequenced. Sectors corresponding to the -35 and -10 regions of the promoter, as well as the beggining of the DsRedExpress-a coding region are underlined. The promoter sequence matches exactly to the *Escherichia coli rrnB P1* promoter

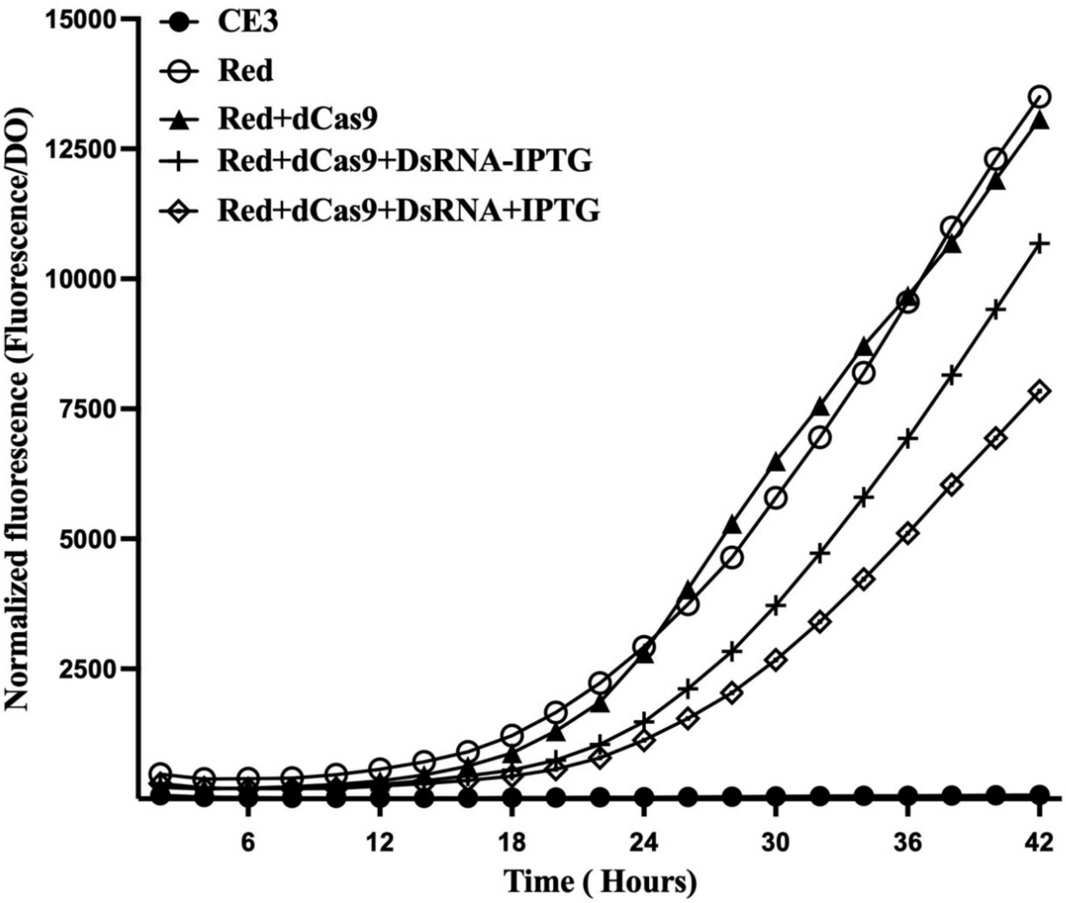

Supplementary Figure 2. Normalized fluorescence of selected strains along the growth curve. Area Under the Curve (AUC) was calculated using GraphPad Prism software for each strain. First, we calculated the AUC for each biological replicate. We then represented the resulting mean AUC of the three biological replicates. Data of AUC are depicted in Figure 2C in the main text.

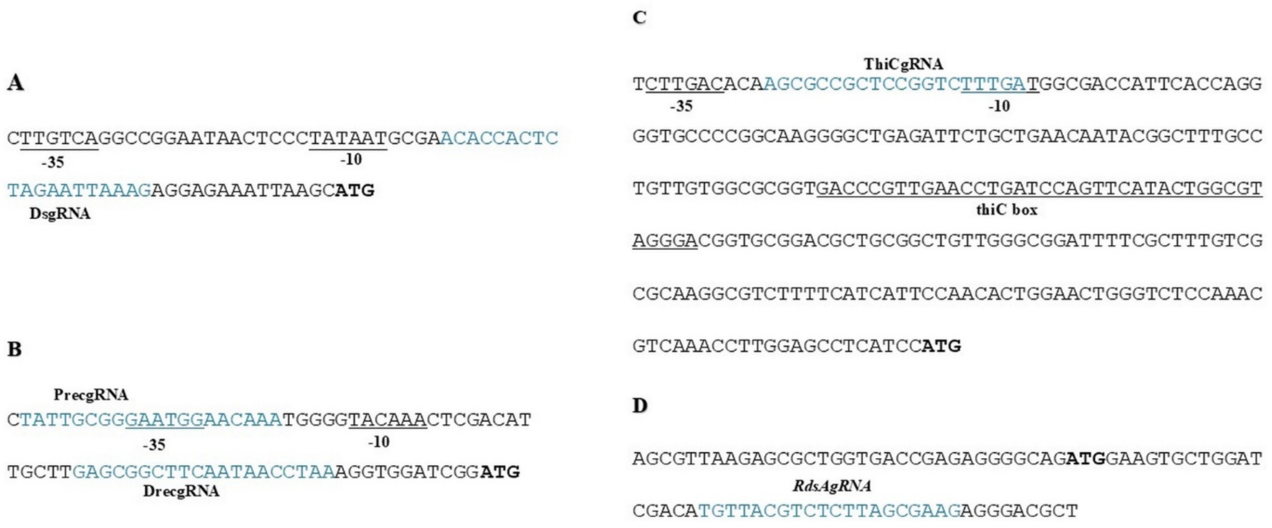

Supplementary Figure 3. Schematic representation of the CRISPRi-targeted sequences. Promoters and other transcriptional regulatory elements are marked and underlined, sequences complementary to guide RNAs are shown in blue, and start codons are indicated in bold. (A) *DsRedExpress* gene. (B) *recA* gene. (C) *thiC* gene. (D) *rdsA* gene (promoter has not been identified).

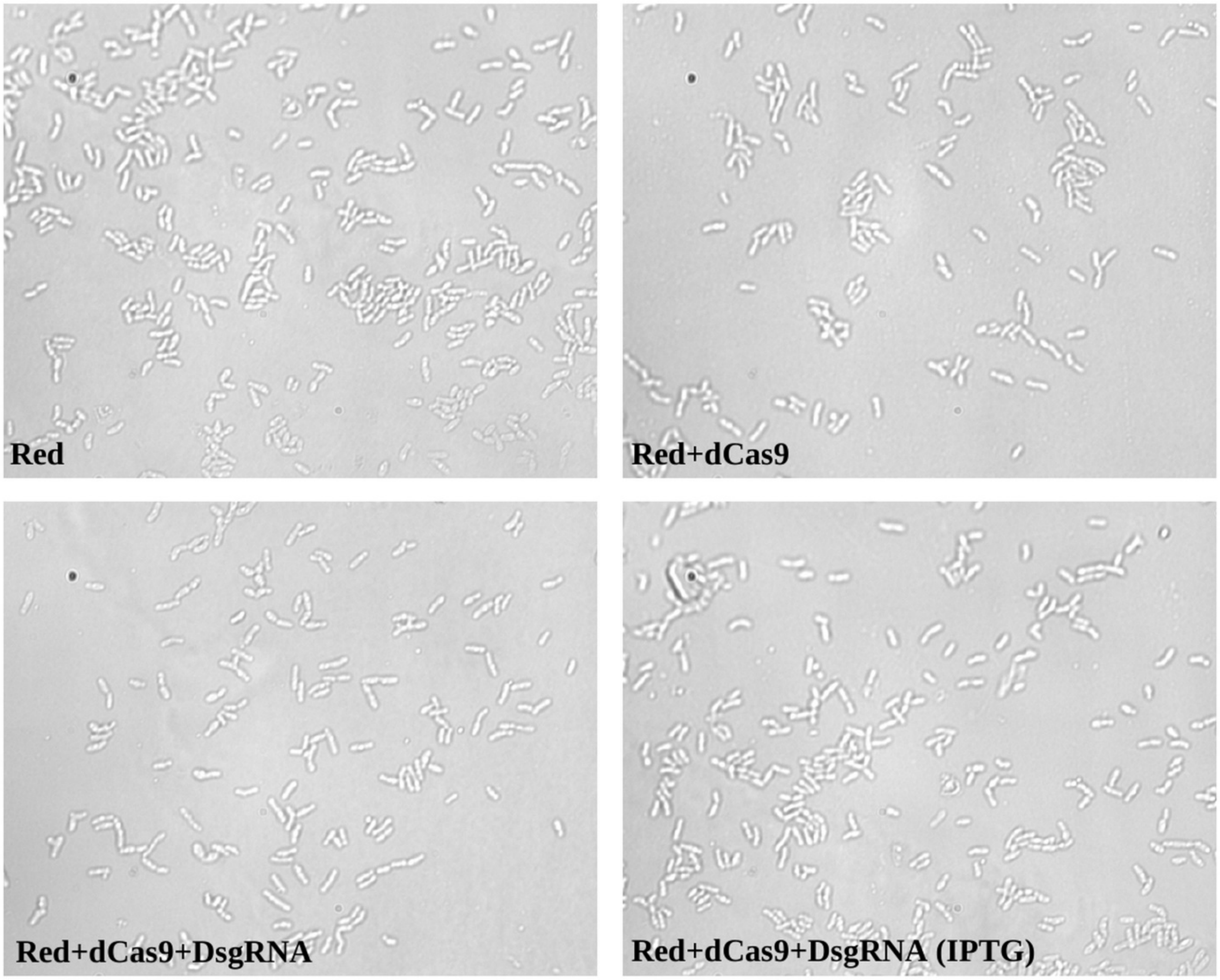

Supplementary figure 4. Expression of dCas9 do not affect cell morphology. Cells of the indicated *R. etli* strains were grown for 12 h on MMY liquid medium and analyzed by light microscopy. Addition of the inducer (IPTG) is indicated. At least one thousand cells were analyzed for each strain. Representative images are shown.

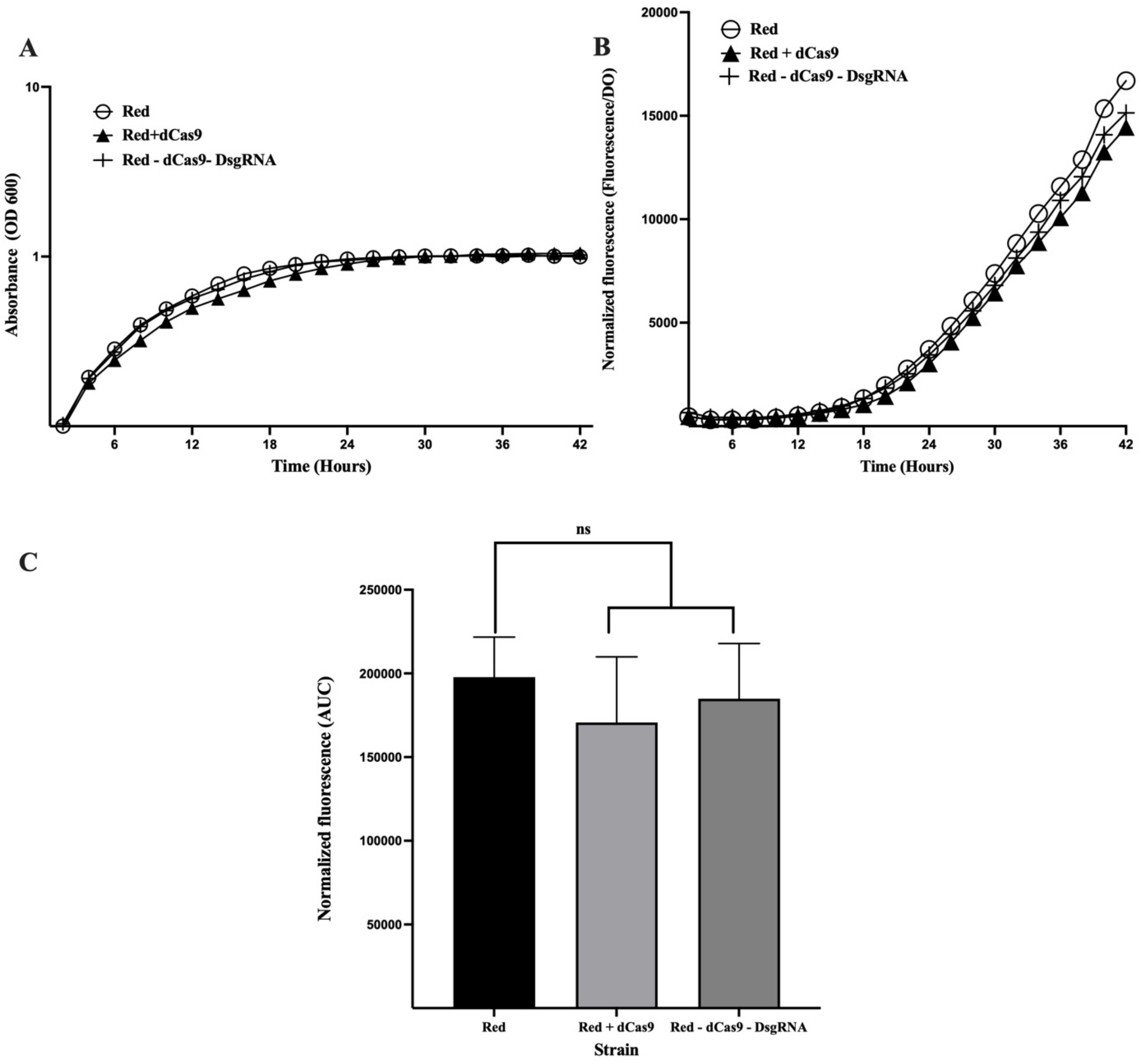

Supplementary Figure 5. Recovery of red fluorescence upon elimination of plasmids harboring dCas9 and DsgRNA. A, growth curves for selected strains. B, Normalized fluorescence of selected strains along the growth curve. Area Under the Curve (AUC) was calculated using GraphPad Prism software for each strain. First, we calculated the AUC for each biological replicate. We then represented the resulting mean AUC of the three biological replicates. Data of AUC reveal no significant differences (ns, p value > 0.05) in fluorescence.

Supplementary Table 1. Strain list

Species and Strains	Plasmid or insertion	Resistance[a]	Source[b]
Rhizobium etli			
Red	mini Tn7 (Gm) $P_{A1/04/03}$ DsRedExpress-a	Gm Fos Nal	Torres-Tejerizo et al., 2015
Red/dCas9	pBBR1MCS-2dCas9	Gm Fos Nal Km	This work
Red/dCas9/DsgRNA	pBBR1MCS-2dCas9 pRhigRNADsgRNA	Gm Fos Nal Tc Km	This work
CE3	None	Nal Fos	Noel et al., 1984
CE3/dCas9	pBBR1MCS-2dCas9	Nal Fos Km	This work
CE3/dCas9/DrecgRNA	pBBR1MCS-2dCas9, pRhigRNADrecgRNA	Nal Fos Tc Km	This work
CE3/dCas9/PrecgRNA	pBBR1MCS-2dCas9 pRhigRNA PrecgRNA	Nal Fos Tc Km	This work
CE3/dCas9/ThiCgRNA	pBBR1MCS-2dCas9 pRhigRNAThiCgRNA	Nal Fos Tc Km	This work
CE3/dCas9/RdsAgRNA	pBBR1MCS-2dCas9 pRhigRNARdsAgRNA	Nal Fos Tc Km	This work
Escherichia coli			
S17	Chromosomal integration of [RP4-2 (Tc::Mu) (Km::Tn7)]	Sp	Simon, 1983
S17/pBBR2	pBBR1MCS-2	Km Sp	This work
S17/pRK415	pRhigRNA	Tc Sp	This work
S17/dCas9	pBBR1MCS-2dCas9	Km Sp	This work
S17/DsgRNA	pRhigRNADsgRNA	Tc Sp	This work
S17/DrecgRNA	pRhigRNADrecgRNA	Tc Sp	This work
S17/PrecgRNA	pRhigRNA PrecgRNA	Tc Sp	This work
S17/ThiCgRNA	pRhigRNAThiCgRNA	Tc Sp	This work
S17/RdsAgRNA	pRhigRNARdsAgRNA	Tc Sp	This work

[a] Fos, fosfomycin; Gm, gentamicin; Km, kanamycin; Nal, nalidixic acid; Sp, spectinomycin and Tc, tetracycline.

[b] Torres Tejerizo G, Bañuelos LA, Cervantes L, Gaytán P, Pistorio M, Romero D, et al. Development of molecular tools to monitor conjugative transfer in rhizobia. J Microbiol Methods. 2015 Oct;117:155–63.

Noel KD, Sanchez A, Fernandez L, Leemans J, Cevallos MA. Rhizobium phaseoli symbiotic mutants with transposon Tn5 insertions. J Bacteriol. 1984 Apr;158(1):148–55.

Simon R, Priefer U, Pühler A. (1983). A broad host range mobilization system for in vivo genetic engineering: transposon mutagenesis in gram-negative bacteria. Nat Biotechnol 1, 784–791. doi: 10.1038 /nbt1183-784

Supplementary Table 2. PCR primers[a]

Primer ID	Sequence	Restriction site	Use
M1	AAAGGTACCATGCTTGCCCG	KpnI	Mega primer with ruvC mutation
M2	CCGATG**G**CCAGGCCGATGCTGTACTTCTTG		
Mega	AAAGGTACCATGCTTGCCCGCGCACACACAGGAATCTATAATCGGGCCGCTGGAGACAGCGCATGGACAAGAAGTACAGCATCGGCCTGG**C**CATCGG	KpnI	nCas9 fragment with ruvC mutation
M3	TGGTCGACGTCGTAGTCGCTCAGGCGGTTGATGTCCAGTTCCTG	AatII	
M4	TACGACGTCGAC**GC**CATCGTGCCGCAGAGC	AatII	dCas9 fragment with HNH mutation
M5	GCTTTCTCGAGGATCTCAGTCGCCGCCCAG	XhoI	
Fw-red	CATTGACCGTTCCTTCCATG		Sequencing of the promoter of DsRedExpress-a
Rev-Gm	TGGCTCTCTATACAAAGTTG		
Ar1	GTCGATCTGCAGAACCTCCT		RT-qPCR-*recA*
Ar2	TCACCATGCAGTTCGACTTG		
Th1	GCGTGACATAGCAGAGCATC		RT-qPCR-*thiC*
Th2	AGGTGATGATCGAAGGTCCC		
Red1	GTTACAGTAACGACGCCACC		RT-qPCR-*dsRed*
Red2	ACGAAGGCCACAATACCGTA		
Rds1	GCAGGATCTCTCGCTGACAT		RT-qPCR-*rdsA*
Rds2	GAGGTTTCGATCGTGGTGAG		

[a] All primers are shown in a 5´-3´orientation. Underlined, restriction sites; bold, mutated bases.

Supplementary Table 3. Oligonucleotides used for cloning of guide RNAs[a].

Guide RNA	sgRNA sequence with the cloning sites	PAM sequence[b]	Target
DsgRNA	**ACGC**ACACCACTCTAGAATTAAAG<br>**AAAC**CTTTAATTCTAGAGTGGTGT	AGG	Downstream the Red protein promoter
PrecgRNA	**ACGC**TATTGCGGGAATGGAACAAA<br>**AAAC**TTTGTTCCATTCCCGCAATA	TGG	Promoter of *recA*
DrecgRNA	**ACGC**GAGCGGCTTCAATAACCTAA<br>**AAAC**TTAGGTTATTGAAGCCGCTC	TGG	Downstream the promoter of *recA*
ThiCgRNA	**ACGC**AGCGCCGCTCCGGTCTTTGA<br>**AAAC**TCAAAGACCGGAGCGGCGCT	TGG	Promoter of *thiC*
RdsAgRNA	**ACGC**TGTTACGTCTCTTAGCGAAG<br>**AAAC**CTTCGCTAAGAGACGTAACA	AGG	Coding sequence of *rdsA*

[a] Oligonucleotides are shown in a 5´-3´orientation. Pairs of oligonucleotides were annealed to generate the desired guide RNAs. Bold, bases added complementary to ends generated by BbsI restriction.

[b] PAM sequence downstream of the region of the guide RNA.
